# Addressing the Mental Health Needs and Building Resilience of Populations Affected by the Earthquakes in Turkey and Syria: Lessons From Haiti and Elsewhere

**DOI:** 10.3389/ijph.2023.1605986

**Published:** 2023-03-28

**Authors:** Jude Mary Cénat, Daniel Derivois

**Affiliations:** ^1^ School of Psychology, University of Ottawa, Ottawa, ON, Canada; ^2^ Department of Psychology, Université Bourgogne Franche-Comté, Dijon, France

**Keywords:** earthquake, Turkey, Syria, mental health needs, resilience

Since the earthquakes in Turkey and Syria, many researchers have published insights on how to address the mental health needs of affected populations (1–3). After reading the ideas proposed with great interest, we found it necessary to provide evidence-based suggestions on how to address the mental health needs of earthquake-affected populations in Turkey and Syria, based on 13 years of intensive research on the 2010 earthquake in Haiti and observations made elsewhere (4–15). Although the social, political, economic, cultural, and religious contexts are different, we have identified overarching aspects and steps that can help to prevent mental health problems among populations affected by natural disasters and build resilience. In the case of the earthquakes in Turkey and Syria, these steps can help professionals, governments, communities, and international organizations better channel resources to build resilience in affected populations.

While interesting, the steps proposed by different researchers remain insufficient (1). Moreover, contrary to what they proposed, to better help prevent mental health problems, the primary concern should not be mental health itself. Affected populations are not ready to receive care in the moments following a natural disaster. Instead, the focus should be on the concrete and physical aspects of meeting the basic needs of survivors. Our research, based on both the mechanisms underlying mental health problems related to natural disasters and those related to building resilience, has allowed us to develop the following seven steps:

Step 1. *Ensure that basic needs are met for all:* food, clean water, shelter, and security (avoiding psychological, physical, sexual, and community violence). As soon as possible, government structures must help reopen schools (even in temporary shelters) and help adults find a job (temporarily, even in a field other than their skills). This first step aims to help the affected populations regain some normalcy in their daily lives.
Step 2. *Emphasize the collective dimension of the suffering and grief:* symbolize the suffering and grief at a collective level by having national celebrations. In the beginning, this can be done by lighting candles every week at the time of the earthquakes, making nationwide gestures that connect people and helping the relatives of the victims realize that these are not individual deaths but national deaths that the whole nation will remember. In addition, the state can organize a national mourning, create a list of victims, and announce a monument that will be erected in a year for the victims while respecting religious aspects. It is a way to give a dignified burial to the missing people who will never be found and to the burials that defy the religious and cultural rites and rituals of the countries (16). This collective mourning is a guarantee for individual mourning.
Step 3. *Massive training in psychological first aid:* enable nurses, teachers, and religious and community leaders to provide active listening to those affected, recognize those in need of professional assessment and/or care, and know where to refer them when necessary. It would be better to advocate for a much more substantial 5-day training to increase the capacity of these professionals and better help them recognize their limitations (17).
Step 4. *Take national action to build collective resilience:* for children, this can be done through arts, sports, reading club, and developing art projects involving parents. For adults, developing community projects, creating spaces to connect, recharge, pray, and listen can help. To help build collective resilience, it is also necessary to allow affected populations to participate in reconstruction efforts, to listen to them, and to not do for them but to do with them. NGOs must join local organizations and avoid any condescending attitude.
Step 5. *Create one-stop ambulatory clinics:* creating outpatient clinics that can reach people and facilitate care is crucial. Having a one-stop care where there is a physical health assessment, application of dressings, and if needed, a referral to psychiatric or psychological care is necessary to avoid people already affected having to take multiple steps to access care.
Step 6. *Create collaborative and short-term care:* developing a one-to three-session care to help people talk about their mental health concerns can help prevent post-traumatic stress, anxiety, and mood disorders, among others. These are not debriefing sessions but sessions that can help those who urgently need to talk to do so in a safe psychological setting. If resources are lacking, 90-min group sessions may be an excellent way to address individual needs. Remote care is also an option to consider.
Step 7. *Protect the children:* it has been shown that after natural disasters, children are more likely to experience different types of abuse and an increase in sexual violence (18–20). Therefore, in addition to protecting children from traumatic images, urgent steps must be taken to raise awareness among parents and affected populations to recognize and report the risks of child sexual abuse.

There are the seven steps that will contribute to reduce the pressure on health services, help prevent the mental health impacts in affected populations, have therapeutic effects, and help build collective resilience to cope and, above all, bounce back. These steps should be integrated into natural disaster preparedness plans. They should be undertaken as soon as the moment of stupor has passed so that rescue actions do not contribute to further traumatizing the affected populations but that at each step, everything is done to help children, adults, and families affected to rebuild.
